# The Botanical Text-Book

**Published:** 1858-01

**Authors:** 


ART. II.—1. *The Botanical Text Book, an Introduction to Scientific Botany.* By ASA GRAY, M.D., Fisher Professor of Natural History in Harvard University. Fifth Edition. New York. Putnam & Co., 1857.

2. *First Lessons in Botany.* By ASA GRAY, M.D. New York. Putnam & Co., 1857.

3. *Genera of the Plants of the United States; Illustrated by Figures and Analyses from Nature.* By ISAAC SPRAGUE. With descriptions by Prof. ASA GRAY, M.D. New York. Putnam & Co. Vols. I. and II., 1849.

4. *Manual of the Botany of the Northern United States.* By ASA GRAY, M.D., &c. Second Edition. New York. Putnam & Co., 1856.

DURING a conversation which we held with Sir William Hooker, the Royal Botanist of England, and a man whose name is as firmly interwoven with the knowledge of the science of plants as that of Linnæus, Jussieu, or De Candolle, the subject falling on our American philosophers, we alluded to Professor Gray. "Gray," said he, "is a man of whom any university, any country, might well be proud. Few men have ever equalled him in scientific research, or excelled him in philosophic deduction." And any one will agree with us in considering this praise well merited, on studying the works whose titles stand at the head of the present article; or the numerous, profound, and invaluable contributions which are published by the Smithsonian Institution, the Federal Government, and Silliman's Journal, from the pen of the same author. The first, third, and fourth of the works before us have a world-wide reputation: no botanical library is complete without them; no conclusions on the Natural History of our continent can be drawn without their aid.

*The Botanical Text Book*, now in its fifth edition, is the most complete treatise on Botany ever issued from our press. There is no work we can more confidently recommend to the use of our colleges and higher academies than this; there is none from which the practitioner will more readily glean the information he desires, or which the mere amateur will find more pleasure in perusing. As a proof of the high distinction in which this work is held abroad, we may state that for some years it was used as the class-book in the University of Edinburgh, (a compliment seldom paid to American literature, but which, we are glad to notice, has recently been extended to Dr. Wood's *Practice of Physic*,) and was only deposed when Professor Balfour, of that city, produced his own elaborate and massive treatise: a book which, however excellent as an encyclopædia of the science, and although got up in a most elegant and attractive style, wants the clearness and brevity which is so remarkable in Dr. Gray's works. The *Botanical Text Book* treats of three of the great divisions of the study,—Structural, Physiological, and Systematic Botany. These are fully and ably entered into. We cannot help regretting, however, that the author omits entirely the two vast subjects of Geographical and Palaeontological Botany. The student should certainly become familiar with these. True, geographical botany is an immense subject of itself, and to be fully discussed would occupy far more space than is contained within the limits of the *Text Book;* but a few general facts should have been inserted, pointing out the general laws respecting the different amounts of temperature, light, and moisture which give rise to the many changes in the Flora from the Equator to the Poles. And this subject of geographical botany possesses no small interest to the medical man, who, by a knowledge of it, might judge *a priori* of the nature of a climate from seeing its flora; the agriculturist, from the same data, may be led to judge of soils; the traveller recognizes his altitude in mountainous countries, and the explorer his longitude, while numerous facts on very various subjects are opened up to us. The subject of fossil or palaeontological botany always deserves notice, bearing the close relation it does to the existing state of the science, and as affording, in many instances, a clue to the development of great and valuable truths.

We are especially glad to notice that Professor Gray has lately sent out into the world an abstract of his *Text Book*, under the name of *First Lessons in Botany*. We say we are glad—we are more—for we feel sure that the intrinsic merit, the general completeness, and the cheapness of this work will of themselves act as a recommendation to it, and strike a deathblow at the existence of the numerous worthless treatises which every now and then are issued from the press to satisfy the pride and line the pocket of some ignorant and needy schoolmaster. The *Outlines* is intended as a class-book for beginners, in which the general principles of the science are not only rendered as simple as possible, but are also sufficiently enlarged upon, forming a complete grammar of vegetable physiology and taxonomy; and indeed, as far as regards the physiological part, the work is complete in itself, and taken in conjunction with the *Manual of the Botany of the Northern United States*, forms a complete library for the student of the botany of this part of our country,—the *Outlines* being to the *Manual* what the grammar and dictionary are to the classical author. The value of the *Outlines*, too, is considerably increased by the addition of a Glossary, which, besides its completeness, gives the proper accent in all instances where there might be a doubt on the subject. The illustrations in both the above works are remarkable for their accuracy and beauty—an accuracy which, so beautifully shown in the *Genera of the Plants of the U.S.*, has given Mr. Sprague the well-earned title of the most accurate of living botanical artists.

The second edition of the *Manual of the Botany of the Northern States* is also a welcome visitor. The first edition, which was more hastily written to supply a pressing want, has been almost wholly and carefully rewritten; the old plan has been preserved, but the work is a new one. Its increased size is mainly owing to the increase of the geographical area comprised in the work, as that now includes Virginia and Kentucky to the south, and passes as far west as the Mississippi River. This area gives a much more natural division to the country. The southern line divides the flora of the cooler temperate from the warm temperate region, as, except on the low coast of Virginia, very few characteristically southern plants occur north of this line. The western limit, too, while it includes some prairie vegetation, yet excludes the plants which are nearly peculiar to our great woodless plains in

Iowa and Missouri. The northern boundary, that of the United States, varies through about five degrees of latitude, and nearly embraces Canada proper, excluding the more northern Labrador flora. Although not passing beyond those limits, considerable value is added to the work by certain marks used in pointing out the distribution of the species within these limits; and to facilitate the comparison of our flora with that of Europe, the mark "Eu." is appended to the names of species occurring in both countries. Another important feature is a series of fine plates of the genera of the Cryptogamous orders of plants, six of which are by Mr. Sprague, and the other eight, illustrating the mosses, by Mr. Sullivant, who is the author of that portion of the work describing the mosses and liverworts. We believe no country can boast of a better "Flora" than the above; few can equal it. Our country contains many great minds in Botany as well as the other sciences, and where the names of Gray, Torrey, and Darlington are known, they are respected as belonging to three masters in botanical science.

We believe we have said enough on this head, and we cannot praise more than we have done. Excellence will find its own without a trumpet being blown before it; but to those desirous of pursuing the most charming of all scientific studies—a science which contributes to expand the mind, cultivate the reasoning faculties, perfect the power of observation, strengthen the body, and assist us in other sciences by pointing out the reasons for things—we would say use these works; put full confidence in them: you can find no better. And who does not wish to study botany? Every lover of nature, every one looking up through nature to nature's God, must desire to know more of His works, to know more of the lovely beauties which surround us at every step; every searcher after truth must desire to penetrate into the mysteries of those phenomena which modern science has revealed to us in the plant-life, a knowledge of which in the plant leads to the understanding of similar wonders in the animal world—ay, in man himself;—

"For in all places and in all seasons
Plants expand their light and soul-like wings,
Teaching us by most persuasive reasons
How akin they are to human things."

But too many are apt to be scared at the very name of botany—to associate its existence alone with a number of long and hardly-pronounceable names, with pages of dry detail and columns of barren facts. But it should not be so. True, there are many long names, but the mastering of these does not constitute studying botany. We have seen many a gardener or mountain guide who could give strings of hundreds of long names, and point out the plants to which they belonged, and yet know nothing of botany. Botany is a true science; the retaining of long words is a mere exercise of memory. These words are merely useful as the representatives of ideas, as the representatives of certain combinations of qualities, to which, for the convenience of scientific men, the Latin or universal language is applied, and by the use of which we point out more certainly what we mean; but they are merely names, bearing the same relation to botany that the name of an individual does to his mind. Botany is the science which treats of plants, of those first elements of life which clothed the fiery mass of this world when it originally cooled; which tells us the history of the tiny forms which first clothe the rugged rocks of the ice-bound Polar regions, and even the snow itself; of those majestic giants, like the banyan of Nerbuddah in India, a single tree of which forms a forest, under whose shade thousands of troops have rested; of the solitary residents on the isolated spots of the burning sands of Africa, storing up moisture for their own support; of the gorgeous carpets of the Western prairies and Southern llanos; of the little blue-eyed gentian, struggling for existence amid the avalanche and storm of the highland Alps; of the mighty pine-tree, with its melancholy roar, in the unexplored Northern wilds; of the tender little pink seaweed, washed on shore by the tide, and perishing ere night; of the mighty Alga, (*Macrocistis*,) riding hundreds of feet along the storm, triumphing amid the uproar; of the little "forget-me-not," raising its meek glance to heaven from out the swamp; and of the gigantic Royal Water Lily, with its flowers a foot and leaves eight feet in diameter; of the microscopic parasite in the human stomach, and of the great Rafflesia, a parasite of Sumatra, whose flower alone weighs fourteen pounds; of the minutest microscopic objects the markings on whose surfaces are only the 1-130,000th of an inch apart; and of the monstrous beds of the coal fields, the forests of a former epoch laid up as fuel for this. Such a variety of interest for study, of opportunity for research, of reason for praise, does botany bring to us! Wherever we go the science may be cultivated. Vegetable life is everywhere; the soil is filled with the embryos of plants ready to burst into existence; the air which covers it is not free from the stores of vegetable life; the waters and rivers abound with it. And such beauty is unfolded to us, such variety of shade, of hue, of form, and perfume! What pleasing associations do the lovely plants not recall! How strange their variety of life, from the little cell which is produced and dies under our observation with the microscope, to the mighty Baobab-tree of Senegal, said to be coeval with Abraham! And does man not owe feelings of gratitude to the vegetable kingdom? Does it not feed him and clothe him, shelter him from the inclemency of the weather, give him relief in pain, solace in misfortune, luxuries and necessaries, pleasures, and education itself? And is the history of these invaluable objects, their growth, development, and peculiarities to be neglected and forgotten, because the completion of the further branches of the study is embarrassed by a few Greek and Latin words? By no means. Let us overcome this small matter and plunge into a new and useful science. Let us consider the science of botany, the phenomena of the growth, life, and death of plants, and the various purposes to which they are applicable, whether in medicine, chemistry, or in any of the different arts and necessities of life. The study of this great subject is too much neglected in the colleges and schools of our country. We are a practical race of people, and are too apt to regard the study of this science as an ornamental rather than a useful pursuit, and thus we teach it generally in our ladies' schools, and send our young women out into the world with a little smattering about "Dicotyledons" and "Monogynias," calling "blue bells" *campanula,* and "chinquapins" *castanea;* but, after all, letting them learn nothing of botany. And the science is practical. The utility and advantage of botanical classification may readily be shown in a variety of circumstances. Structural botany leads to a knowledge of peculiarities, and these peculiarities we are led to classify in various groups or families, in which the genera and species are not only given with the same outward form, but possess similar properties. For instance, let a botanist examine a flower, and, though ignorant of it, he can yet tell from its character where it belongs in the scale of the vegetable kingdom; and whether poisonous, good for food, or otherwise. So also the physician, having by his careful, clear, and undeniable experiments ascertained the medical properties of one plant, will be enabled to determine the virtues of all the others in that genus, or even family.

But it is to medical men that we at present address ourselves, and on them that we would impress the importance of this study. Although we may say it is almost entirely ignored by our medical schools, yet in other countries of older standing it is fully recognized. In France, England, and Germany, the case is very different. By all the medical examining boards of England, except in the very practical examination of the Royal College of Surgeons, which is confined to surgery, physiology, and anatomy, a knowledge of botany, more or less complete, is required. Students are compelled to study it as one of the preparatory branches, and are examined in it with almost as much minuteness as in chemistry. The Boards of the Royal Navy, of the Edinburgh University, and of the Honorable East India Company, are particularly strict on this subject. Consequently all the schools have a teacher of the branch. In the German universities, where all branches are so subdivided that almost any one can be pursued by a student as a specialty, there are generally several branches of the science. For instance, in the University of Bonn, where we spent some of our student's career, Prof. Treviranus gave a course of lectures on the General

Principles of Botany, another on the Natural Families of Plants, and a third on the Peculiarities and Properties of the Flora of the Vicinity; Prof. Brandis lectured on the Anatomy and Physiology of Plants, on the Medical Properties of Plants, and gave a special course on Sleep, and the other Phenomena of Plant Life. In France the method is very similar, and so in other European countries. Here, then, is an open avowal of the importance of the study, and means provided for the full prosecution of it. We have, however, endeavored to reduce our period and amount of medical study to a minimum, and neglect, in consequence, many important branches. And we are not content in so doing. Neglecting botany, we might as well neglect chemistry, for the one is as important a guide to the materia medica as the other. Our knowledge of the properties of plants around us ought to be as great as that of the minerals. We employ a greater amount of botanical medicines, and we are more constantly thrown into situations where a knowledge of the appearance and properties of plants would be of immense practical importance. How many excellent physicians have found themselves at a loss for a remedy, when some old woman of the village came with an unknown and slighted herb which had the desired effect? How many men have become distinguished and rich (we do not allude to quacks, who do, unfortunately, often profane the name of botany) by a mere knowledge of the herbs growing around them? And how many fatal accidents by poisoning have occurred, when the medical attendant did not know that certain herbs produced certain effects, and that antidotes for their poison were within his reach, did he but know what? Our fathers in medicine were all botanists, "herbalists" as we now somewhat contemptuously style them, and they felt the full force and advantage of the study, from the practice of which they gleaned those precious truths which, even now, with our perfections in science, we can only ratify.

The origin of the study of botany is lost in obscurity. The Holy Scriptures and Homer give us the first traces of its study. King Solomon, we are told, in one of his lost books spake of trees "from the cedar tree that is in Lebanon to the hyssop that springeth out of the wall." The Magi, too, doubtless employed the learning acquired by the study as a means of influencing and controlling the unlearned. Asia and Egypt supplied Greece with its beginnings of knowledge, and among the Greeks we find the earliest systematic botanical treatises. Pliny tells that Pythagoras travelled in Egypt, and on his return wrote a work (quoted by Pliny) on the Properties of Plants, derived, doubtless, from the information gleaned during his tour. A disciple of his, Empedocles, wrote the first work on Vegetable Physiology, and in this, amid curious blunders and wonderful theories, numerous outlines of the facts elaborated by modern science are boldly traced. Hippocrates is a name under which several authors wrote about this time, and described in vague and uncertain manner numerous plants. These men, the pioneers of our science, "groping blindly in the darkness," received the name of Rhizotomæ, or "reed-cutters," as their attention was chiefly directed to the medical properties of plants. But the foundation of the great science of botany is attributed to Aristotle of Stagira, who held the difference between the animal and vegetable world to be that plants, though possessed of life, have no innate consciousness or organs of sense to know what is out of themselves, and that they were thus intermediate between animals and rocks. A disciple of his, Theophrastus, who was born in Lesbos, and flourished in the third century B.C., has left behind him a work entitled the *History of Plants*, in which he considers their origin, propagation, and anatomy, and also the phenomena of vegetable life, giving an outline of structure, which, considering his want of magnifying power, is highly wonderful. He distributes vegetables into seven classes, which are characterized by their places of growth, their generation, their use as esculents and potherbs, their lactescence, their size, and the consistence of their sap. The next botanist of consequence was Dioscorides, a Roman citizen, by birth a Greek, who wrote three hundred years after Theophrastus, and described six hundred plants. These he arrayed in four classes, founded on their use in medicine and domestic purposes, viz.—1. Aromatic, 2. Vinous, 3. Medicinal, 4. Nutritious. His labors were so great as to win for him the title of the "Father of the Materia Medica," but the value of his work is greatly diminished from his want of the knowledge of genera and optics. Contemporary with him were Antonius, Cato, Virgil, Varro, and others, who not only showed botanical knowledge, but also a considerable acquaintance with agriculture and rural economy. Pliny the elder described about one thousand different species of plants, but, like other ancient botanists, he scarcely used any other mode of arrangement than the general division into trees, shrubs, and herbs. Galen stands next as an author on Medical Botany, and after his time, except by the Arabians, who seem to have paid some attention to this branch, botany, with the other sciences, was neglected. Here and there some monk cloistered in a sequestered vale may have passed his tedious hours in such employment, but to us, on looking back, all is blank till the revival of letters in the fifteenth century, when literature had its shelter in Italy, and the works of ancient authors were republished, translated, and commented on. America was discovered, Marco Polo, of Venice, examined the treasures of Middle and Southern Asia, and Eastern Africa, and described plants from India and the Indian Sea. From that time till now, the science has progressively advanced, first, in the addition to our knowledge of the names, and secondly, of the structure and physiology of plants. Italians, Spanish, Portuguese, and Germans assisted in increasing and perfecting our knowledge, and in the seventeenth century we find the pursuit of botany fairly recognized as an independent science and study. We find now the formation of a greater number of medical schools and universities; and to show the importance of botany at this time, it would appear from all authentic documents that the first medical chair in the University of Edinbugh was that of Dr., afterward Sir Robert Sibbald, (1685,) who not being confined to any particular branch, and constituting in himself the entire Faculty, lectured on Medicine and Medical Botany; and on his subsequently confining himself to the practice of Physic, a second chair was formed by the trustees for instruction in Botany, Dr. G. Preston receiving the appointment in 1706. The whole history of this University goes to prove the general regard and appreciation of the study, and at present the botanical chair there is one of the best-paid positions for a natural historian in the world. From the period above mentioned, Great Britain claims a distinguished share in the honors of botanical discovery. The London Society for the Promotion of Science gave much attention to the subject, and their secretary, Nehemiah Grew, published his famous folio on the *Anatomy of Plants*, in 1682, a work full of new discoveries and laborious examinations. Robert Morison, a Scotchman, professor at Oxford, distinguished himself as a classifier, and formed one of the earliest systems. Shortly after him appeared a yet more distinguished man: John Ray, an English clergyman, a man whom all botanists and naturalists love and honor, enunciated the true principles of classification, and showed the sexual character of plants. With him we find the names of Gerarde, Dr. Hans Sloane, John Parkinson, of Chelsea, and many others, distant but bright stars on our horizon. On the continent, Gesner, Bauhin, Commelin, Rudbeck, and others appeared, and sent down to us, with the results of their labors, their names attached to various beauteous objects in the vegetable world, *monumenta perennia aere.* But we have neither space nor the ability to enumerate and pay individual tribute to the memory of these great men—great physicians who saw the benefit their science acquired by the knowledge of objects around them, and who scorned not to investigate anything however low in the scale of being, which they thought might aid in the furtherance of great truths. We hate to see the finger of contemptuous scorn pointed at such men, their honest labors scoffed, their piety and innocence rendered ludicrous. They are men who deserve our undying gratitude, who have assisted to build up that great temple of our art, which we, in our impatient haste, too often threaten to bring to ruin.

The 23d of May, 1707, is a day long to be remembered in the annals of botanical science. On that day in the humble residence of Nicholas Linné, clergyman of the village of Rooshoolt, in Smaland, a Swedish province, Carl v. Linné, the renowned Linnæus, first saw the light of the world.

"This man," says one of his biographers, "educated in the severe school of adversity, accustomed from his earliest youth to put a high value on verbal accuracy and logical precision, endowed with a powerful understanding, and capable of enduring immense fatigue both of body and mind, produced a most important revolution in botanical science. He improved the distinctions of genera and species, introduced a better nomenclature on the binomial method, and invented a new and comprehensive system founded on the stamens and pistils." This is the man to whom all naturalists bow the knee, to whom botanists owe the present perfection of their knowledge. But we cannot dwell on him as we would willingly do, nor more than allude to the more famous of the rapidly-succeeding line of successful laborers in the field of anatomical and physiological botany:—De Saussure, Duhamel, Link, Rudolphi, Mirbel, Kieser, Schleiden, Mohl, Darwin, and Quekett; in classification, Jussieu, De Candolle, Robert Brown, Sir J. E. Smith, Sir W. Hooker, Lindley, Babington, Torrey, and Gray.

But we consider it both of value and interest to our readers hastily to glance at these men who have paid more especial attention to the botany of our own country, those who, native-born or European, are justly entitled to the name of "American botanists." The best account of these is contained in the *Memorials of Bartram and Marshall*, by Dr. W. Darlington, of West Chester, than whom no living man has more fully identified himself with the botany of his country. American literature has not produced much of importance on physiological botany, but in the record and description of its own flora it has many works of interest and value. Of these we would mention the chief. If not the first, one of the earliest works on North American botany is that of a French botanist, Cornutus, published at Paris in 1635, and entitled *A Description of the Plants of Canada;* the author never having been in America, wrote from dried plants sent home to him. The next is the *New England Rarities* of John Josselyn, an Englishman, who resided nine years in Massachusetts, and published in 1672. He seems to have indulged in no small degree in the marvellous, as he talks of "frogs a foot high," and further states "that barley degenerates frequently into oats." Next we find, in 1680, the Rev. John Banister, who published a catalogue of plants observed by him in Virginia, and who was killed by falling from some rocks in the pursuit of his study. John Bartram, whom we may regard as the greatest of our early botanists, began to collect in or about 1730. He described more plants than any of his cotemporaries, founded the first botanical garden in the country, carried on large correspondence with the naturalists of Europe, and proved himself a shrewd and profound observer. Catesby, Clayton, Colden, Logan, Mitchell, Kalm, Kulm, and Garden next appear as workers in the field, and many of our most beautiful and valued plants bear their names as memorials of their labors. In 1773, Humphrey Marshall, another illustrious self-educated native botanist, established the second botanic garden in the village now called Marshalton, Chester county, Pa., where most of the trees remain to this hour noble specimens of his gardening skill. We fain would dwell among these noble pioneers of our science, for we feel it no fit usage of such men to pass them by with mere mention of their names. But we are compelled to use them thus rudely. Marshall's work on the forest trees of America, his *Arbustum Americanum*, may be called the first strictly American botanical work, that is, written by a Native American, and published in the country, and it does both much credit. The first work on American Materia Medica was published at Erlangen, in 1787, by Dr. Schoepf, a German who had lived some time in the United States. And now we come to names more familiar to us,—those of men who are themselves alive, or whose pupils are around us: Prof. Barton, who published the first elementary botanical treatise in this country; the two André Michaux, whose works on our oaks and forest trees form the greatest mass of our knowledge on the subject; Rich, Muhlenberg, Major John Le Conte, Thomas Nuttall, Stephen Elliot, and Frederick Pursh, all of whom have produced excellent and valuable works on special or general floras. In 1817 an eccentric naturalist, Rafinesque, published his first work on the flora of Louisiana. Of him, however, it may be said that he made such unreliable statements, and introduced such startling innovations in nomenclature, that although he possessed undoubted botanical knowledge, yet his works were rather prejudicial than beneficial to the science. *Silliman's Journal*, the Proceedings of the Philadelphia Academy of Sciences, and Torrey's first works appeared about 1820, and from this period till now, general and local floras have increased and multiplied. Eaton, Torrey, Sir W. Hooker, and Drs. Beck and Gray have given us the best and most reliable of these. Different districts of our country have been explored by different authors, and numerous monographs by various distinguished botanists have contributed valuable additions to our knowledge of the distribution of families and genera. Thus we are indebted to Dr. Torrey for a flora of the Rocky Mountains, to Dr. L. Beck for a flora of Illinois and Missouri, Dr. Brereton contributes the flora of the District of Columbia, Aikin supplies that of Maryland, and Croom of North Carolina. In 1837, Dr. Darlington published his *Flora Cestrica*, now in its third edition, and probably the best local flora ever published. Mr. Sullivant, Mr. Thomas Lea—the lamented brother of the eminent conchologist of this city—and Prof. Riddell, of New Orleans, have opened up the flora of Ohio and the Western Country. For a knowledge of the plants of Milwaukie, we are indebted to Mr. Lapham; of Delaware, to the Botanical Society of Wilmington; of Rhode Island, to Mr. Olney, of Providence; of Florida, to Dr. Chapman; of Louisiana, to

Prof. Riddell; of Missouri, to Prof. Engelmann; of Kentucky, to Dr. Short; of Massachusetts, to Prof. Bigelow and Mr. Emerson; and for the magnificent flora of New York, in two quarto volumes, published by the State, to Dr. Torrey. And yet though all this is done, much remains unknown, unexplored. Numerous workers in the fields will yet find their labors amply repaid by pursuing their investigations in our great territory. Many of the States are without their written floras; much remains to be opened up, many wondrous facts to be developed, many valuable qualities to be redeemed from obscurity. Our medical men ought particularly to bear this in mind. Some of our drugs are becoming rarer and more expensive in the market. Why? Not certainly because they are being used up, or are dying out before the progress of cultivation, but because the aborigines who knew these plants and collected them for our markets are themselves driven back and their successors recognize not the herbs which surround them. Since the days of Schoepf, some others have pursued the subject of the medical botany of this land. Barton, Rafinesque, and Bigelow have produced works on this important subject, and now our great lawgiver in medical matters, the American Medical Association, strongly recommends the study, and in its Transactions gives us noble trophies of what it has done; inspiring us with desire to follow the example. Turn over your volumes and look for yourselves. "Dr. Clapp on the Medical Flora of the U.S.," "Dr. Peyre Porcher on the Cryptogamic Botany of the U.S.," and others, are noble specimens of labor for the love of science and improvement of the co-operators.

We have already alluded to the importance of the study of botany by medical men desirous of understanding their profession. But it may be urged that in acquiring a knowledge of materia medica a sufficient acquaintance with botany is obtained. This we so far allow. Sufficient indeed to mix a pill or powder, sufficient for an elegant prescription, sufficient perchance for distinguishing between good and bad drugs; but sufficient to allow the physician to trust to his own resources in procuring a remedy, to emulate our fathers in medicine in their laborious experiments on which our knowledge is founded, we deny. And we Americans are somewhat in the position of these ancient fathers. With a wide and almost unknown territory full of vegetation, with new diseases springing up around us, and unknown and unexamined herbs amid our forests, and with the ancient remedies of our forefathers lost or of no avail, it is our duty to examine and to go on examining, experimenting, heaping up facts, with faith, patience, and perseverance, until we have gained a knowledge of all. We have heard of a theory that no disease exists in a country without an antidote springing up from the soil, and Scotch boys tell us "no stinging nettle grows without a dockin leaf close by," and we believe there is much truth in the statements. We are governed by an allwise Providence, whose desire is for our good, and who, while He gives us the means, requires us to discover and adopt them.

It is not, however, merely as an aid to the discovery of remedies for disease that we would urge the study of botany on medical students. We would recommend it to them as a primary means of mental discipline, holding the same relation to their future professional career as the study of the classics does to the development of the mind of the educated orator and statesman. The habits of observation are encouraged, the attention is arrested, the judgment brought into action, the logical powers strengthened, and method acquired by the pursuance of this branch. Then, again, it is in the vegetable kingdom that we find the first and simplest application of those great laws which govern life: in the vegetable cell we recognize the representative of that cell from which we ourselves, thinking, intelligent beings, are produced; in the vegetable phenomena we find a clue to many of the higher and more complex phenomena of the animal kingdom. And in the study of microscopic botany, the labors of Robin, Leidy, Goodsir, Ehrenberg, Berkeley, and others have shown us the importance of recognizing vegetable life, as it is distinctly proved that various diseases owe their origin to vegetable spores, a fact of monstrous importance when we come to treatment.

Besides all this, botany exerts another claim for attention on the medical man, and this is in the study of the laws of hygiene—the examination of the great laws under which the vegetable world exerts its influence on the atmosphere, by moistening or cooling it, by filling it with poisonous matters or purifying it therefrom. And a knowledge of these facts to the explorer, to the army medical man, to our pioneers in the Western wilds, should be indispensable.

We have ventured to prolong our remarks thus far at the risk of proving tedious, but we would add a few words on the study of natural history generally. To all, but more especially to those of us whose calling is exercised in the country, the study of nature offers the highest attractions. It is the cheapest, most accessible, and, at the same time, the most instructive and delightful of all studies. Its text-book is around us wherever we turn; the world of wonders everywhere holds forth its charms and fascinations—a creation of beauties, abundant, universal, and so adapted to our being that the first impulse of our childhood on being laid on the green sward is to grasp at butterflies or cull wild flowers. And this, the first, the fondest attraction of our natural being, retains, unless contaminated by the allurements of society or cares of business, its freshness to the end. Wealth may become a burden, honor an uneasiness, feasts may pall and friends may fade, but the warm green of the meadow, the soft enamel of the flower, the gentle murmur of the brook, the glorious majesty of the sun, the mellow brightness of the moon, the whole gorgeous panorama of nature remains, and the poor worm of man, who lay withering in art, revives in nature; on the brink of the tomb itself he feels a triumph over death, a consciousness of immortality which misery cannot shake nor skepticism cloud. And if a glance will afford such pleasure, what delights will not the study unfold.

And this study has become among men an honorable one. No more is the simple and enthusiastic naturalist pooh-poohed and scoffed as a madman,—"a harmless being going bug hunting because he has not spirit to follow a fox." No; he sits in high places, honored by all, respected by kings.

Do you seek for pleasure? As a naturalist you find the purest of all pleasures. For wisdom? It is here in store. Let no one view it as effeminate and pedantic. As Kingsley says in *Glaucus*, (and we wish we could quote the whole of his remarks on the subject,) "The qualifications for a naturalist are as many and lofty as were required by old chivalrous writers for the perfect knight-errant of the Middle Ages." Every mental, every bodily power is employed, all are used and exercised. What man of us will not gladly pursue this great subject, open for himself the book of nature, and explore that wondrous kingdom—

> "———— where all is formed
> With number, weight, and measure;
> Each . . . . . holds a rank
> Important in the plan of Him who framed
> This scale of beings, holds a rank which, lost,
> Would break the chain and leave behind a gap
> Which Nature's self would rue."

